# Modeling the indirect effect of *Wolbachia* on the infection dynamics of horizontally transmitted viruses

**DOI:** 10.3389/fmicb.2015.00378

**Published:** 2015-04-28

**Authors:** Jakob F. Strauß, Arndt Telschow

**Affiliations:** Genome Evolution Group, Institute for Evolution and Biodiversity, Westfälische Wilhelms Universität MünsterMünster, Germany

**Keywords:** *Wolbachia*, overcompensation, undercompensation, virus, mathematical model, coinfection, life-cycle

## Abstract

Intracellular bacteria of the genus *Wolbachia* are widely distributed in arthropods. There is growing empirical evidence that *Wolbachia* directly interacts with viruses and other parasites inside the arthropod host, sometimes resulting in low or no pathogen replication. Previous theoretical studies showed that this direct effect of *Wolbachia* can result in a reduced virus prevalence (within the population), suggesting that *Wolbachia* could be used in the biological control of vector-borne diseases (e.g., dengue fever). However, *Wolbachia* might also indirectly affect virus dynamics because *Wolbachia*-induced reproductive phenotypes (cytoplasmic incompatibility or male killing) increase the larval mortality of hosts and thus alter the age structure of populations. We investigated this indirect effect using mathematical models with overlapping generations, and found the results to depend strongly on the host's life history. In general, the indirect effect can result in two different outcomes: (1) reduced virus prevalence and virus invasion ability, and (2) increased virus prevalence and virus invasion ability. The former occurs for host species with larval competition and undercompensation, the latter for hosts with either adult competition or larval competition and overcompensation. These findings suggest that the effect of *Wolbachia* on a specific virus is sensitive to the host's life history. We discuss the results with respect to biocontrol programs using *Wolbachia*.

## Introduction

The study of mutualistic and parasitic interactions is a major theme in ecology and evolution (Bourtzis and Miller, 2008; Goater et al., 2013). Most of the past research had a focus on systems with one host and one symbiont species, but there is growing interest in more complex interactions that involve several types of parasites and/or mutualists (Turnbaugh et al., 2007; Rutrecht and Brown, 2008; Rigaud et al., 2010; Knowles et al., 2013). A well-studied phenomenon is that the presence of one symbiont (e.g., a mutualist or mild parasite) reduces the fitness of a second symbiont (e.g., a strong parasite) (Kaltenpoth, 2009). A case in point is the intracellular bacterium *Wolbachia*, which impedes replication of dengue virus in the yellow fever mosquito *Aedes aegypti* (Moreira et al., 2009; Bian et al., 2010; Frentiu et al., 2010; Walker et al., 2011). Recently, some projects were initiated that aim to use this effect in biocontrol of vector-borne diseases (www.eliminatedengue.com). In order to evaluate opportunities and potential risks of this new approach, mathematical modeling is useful because it allows investigating factors where pest control is most effective, but also circumstance in which unexpected or counterintuitive effects occur.

*Wolbachia* is a group of alpha-proteobacteria that is widely distributed in arthropods, with 20-70% of insect species estimated to be infected (Werren and Windsor, 2000; Hilgenboecker et al., 2008; Zug et al., 2012). Typically, transmission is maternally from mother to offspring, but there is also evidence for rare horizontal (Werren et al., 1995; Baldo et al., 2006) and paternal transmission (Hoffmann and Turelli, 1988; Nigro and Prout, 1990). *Wolbachia* are well known for the ability to modify the host's reproductive system to their own advantage. The two most common forms of reproductive parasitism are cytoplasmic incompatibility (CI) and male killing (MK). CI-*Wolbachia* induce a sperm-egg incompatibility in matings between infected males and uninfected females, resulting in no or a reduced number of offspring. This allows *Wolbachia* to invade rapidly in host populations as demonstrated by theoretical and empirical studies (Turelli and Hoffmann, 1991; Riegler et al., 2005). MK-bacteria cause the death of infected male hosts, typically during embryogenesis or in early phases of larval development. This is thought to promote the spread of *Wolbachia* if sisters benefit from the death of their brothers, e.g., by reduced sib competition (Hurst, 1991; Hurst et al., 1996). Though CI and MK are very distinctive phenotypes, they share one feature that is crucial for the later analysis. Both alter the age structure of infected host populations by reducing hatch rates or increasing larval mortalities.

A growing number of studies report that *Wolbachia* directly interferes with viruses and other pathogens inside the arthropod host. This *direct effect* of *Wolbachia* can either impede or promote the pathogen's replication and survival (Zug and Hammerstein, 2015). Examples for the former include the West Nile Virus (Glaser and Meola, 2010; Hussain et al., 2013), Dengue Virus (Moreira et al., 2009; Bian et al., 2010), Chikungunya Virus (Moreira et al., 2009), several RNA viruses infecting *Drosophila* (Hedges et al., 2008; Teixeira et al., 2008), and *Plasmodium falciparum* (Moreira et al., 2009). Examples for a neutral or pro-pathogenic effect of *Wolbachia* include *Brugia pahangi* (Dutton and Sinkins, 2005), Japanese encephalitis Virus (Tsai et al., 2006), Drosophila C Virus (Osborne et al., 2009) and *Plasmodium gallinaceum* (Baton et al., 2013).

It is important to note that all mentioned studies report virus prevalence in host individuals or cell lines, but that empirical data for wild populations are lacking. In a theoretical study, Fenton et al. (2011) investigated the tripartite interactions of *Wolbachia*, virus and host. It was shown that the presence of a virus facilitates the invasion of *Wolbachia*, and that the *Wolbachia* spread results in reduced population-wide virus prevalence if *Wolbachia* suppress the virus in coinfected host individuals. More recent work stressed the importance of non-protective symbionts on the epidemiology of pathogens (Ryder et al., 2014). It was shown that the infection dynamics of sexually transmitted diseases of ladybird beetles is significantly affected by the presence or absence of male-killing bacteria in the host. This study showed for the first time that symbionts affect pathogen dynamics *indirectly* by altering the host demography.

In the present study, we considered a scenario where *Wolbachia* and viruses do not interfere directly in coinfected individuals. In contrast to Ryder et al. (2014) we did not investigate the effect of sex ratio distortion, and the virus is not sexually transmitted. Nevertheless, our mathematical model analysis shows that there is an *indirect effect* of *Wolbachia* on the virus dynamics. The indirect effect occurs because both, CI and MK infections increase the average larval mortality of host populations. We found that it can either promote or impede virus prevalence, and that the outcome crucially depends on the host's life cycle.

## Mathematical model

**Figure 1:**
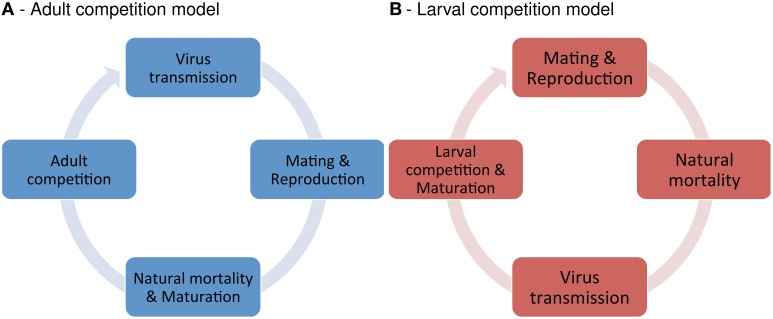
**Model structure**. Illustrated are the life cycles of the adult competition model (ACM) and the larval competition model (LCM).

We designed two basic models in order to investigate the effect of *Wolbachia* on the infection dynamics of horizontally transmitted viruses. The models differ with respect to the host life cycle (see Figure 1). In the adult competition model (ACM), population density is regulated at the adult stage. It describes species where both larvae and adults compete over the same resource. This model may apply to beetles like *Tribolium* sp., which live in colonies with overlapping generations and strong competition between larvae and adults (Costantino and Desharnais, 1991). In the larval competition model (LCM), density regulation occurs during larval development. Here, larvae and adults are assumed to exploit different resources. The LCM allows further to distinguish between over- and undercompensation. For the former, larvae reduction results in an increase of maturing adults, whereas the latter shows the opposite effect. The LCM may apply to mosquitoes and other dipteran. E.g., yellow fever mosquitoes (*Aedes aegyptii*) and related species lay eggs in small breeding containers, in which larvae compete for resources. This results in scramble competition amongst larvae. However, resource competition between adults is generally thought to be negligible or absent in this system (Dye, 1984).

Virus transmission in both models happens between adults and follows the mass-action principle. Infected individuals suffer once a reduced survival probability and act as virus reservoir if they survive. Curing is not possible. Though both models describe the virus dynamics among adults, they differ with respect to the type of variables used. The ACM describes the temporal change of the infection frequency. This approach is justified because density dependent regulation happens in the adult stage, which keeps the adult population size constant through time. The LCM, however, keeps track of the absolute numbers of infected and uninfected individuals. A simple “infection frequency model” cannot be used because density regulation is in the larval stage, whereas virus transmission happens among adults.

An important feature of both models is that they allow for overlapping generations and distinguish between larval and adult stage. This is in contrast to previous theoretical work on *Wolbachia* and viruses (Fenton et al., 2011). Note further that the two basic models do not explicitly include *Wolbachia*. Instead, the effect of *Wolbachia* is implemented as a reduction in the average larval mortality of the host. This allows comparing *Wolbachia*-infected and uninfected populations by varying a single parameter. Biologically, this approach is justified because both, CI and MK, increase the average larval mortality in *Wolbachia*-infected populations. Complex models involving explicit CI and MK dynamics were also analyzed, but revealed qualitatively similar results (see discussion and supplementary for details).

### Adult competition model

The adult competition model (ACM) describes the temporal change in the infection frequency of the virus with a deterministic non-linear recursion equation. During each time step, the following events occur in order: (1) virus transmission and virus-induced mortality, (2) mating and reproduction, (3) natural death of larvae and adults, and (4) density dependent population regulation (see Figure 1A). The population has the constant size *N* at the beginning of each time step. Let *X* denote the virus frequency of adults at this point. In order to calculate the virus frequency in the next time step (*X*′) each phase of the life cycle is modeled separately.

First, virus transmission takes place. We assumed that each individual has random contact to one other individual. If an infected and an uninfected individual meet, transmission occurs with probability *t*_*V*_. The newly infected individuals suffer once a reduced survival probability of (1 − *s*). Note that individuals, who were already infected, do not suffer from the pathogen and have the same survival probability as uninfected individuals. As a result, the population consists of *i* = [*X* + *t*_*V*_(1 − *s*)*X*(1 − *X*)]*N* infected and *u* = [(1 − *X*) − *t*_*V*_*X*(1 − *X*)]*N* uninfected individuals.

Next, mating and reproduction happen. We assumed that each individual lays ε eggs that all develop into larvae. Adults and larvae face natural mortality, i.e., the fraction *d*_*A*_ of adults and *d*_*L*_ of larvae die. All surviving larvae develop into adults. The resulting population consists of (1 − *d*_*A*_) *i* infected and (1 − *d*_*A*_) *u* + ε(1 − *d*_*L*_)(*i* + *u*) uninfected adults.

Finally, density dependent regulation occurs. Mathematically, this is realized by normalization. The infection frequency in the next time step is calculated as the number of infected individuals divided by the total number of individuals. This yields the following recursion equation:

(1) $$X^{\prime }=\frac{\left(1-d_{A}\right)\left[1+t_{V}\left(1-s\right)\left(1-X\right)\right]X}{\left[1-d_{A}+\varepsilon \left(1-d_{L}\right)][1-st_{V}X\left(1-X\right)\right]}.$$

### Larval competition model

The larval competition model (LCM) keeps track of the absolute numbers of virus-infected (*I*) and uninfected (*U*) adults in a population. The temporal change of *I* and *U* is described by two non-linear recursion equations. Within each time step, the following events occur in order: (1) mating and reproduction, (2) natural death of larvae and adults, (3) virus transmission between adults and virus-induced mortality, and (4) density dependent regulation of larvae and emerging of new adults (see Figure 1B). *I*′ and *U*′ denote the values of the variables in the next time step.

First, adults mate and produce on average ε eggs per individual. Second, a fraction *d*_*L*_ of the eggs dies during embryogenesis and early larval development. The number of surviving larvae computes to *L* = ε(1 − *d*_*L*_)(*I* + *U*). Further, a fraction *d*_*A*_ of adults dies, resulting in $\tilde{I}$ = (1 − *d*_*A*_) *I* infected and $\tilde{U}$ = (1 − *d*_*A*_) *U* uninfected individuals.

Third, virus transmission occurs between adults. In this model, virus transmission depends explicitly on the number of adult individuals in the population (and not on the infection frequency as in the ACM). We first introduce a contact rate *c* that is linear in the number of infected individuals, i.e., *c* = $\tilde{I}$/*K*. The rate *c* describes the average number of contacts with infected individuals per uninfected individual. The interpretation of *K* is as follows. If $\tilde{I}$ = *K*, each uninfected individual has exactly one contact to one infected individual. For $\tilde{I}$ > *K* there are several contacts, and for $\tilde{I}$ < *K* not every uninfected individual has contact. Next, we calculate the number of newly infected adults. For this, we first calculate the probability of an uninfected individual to stay uninfected within this time step as (1 − *t*_*V*_)^*c*^. Here, *c* stands for the average number of contacts and (1 − *t*_*V*_) for the probability to not get infected despite having contact with an infected individual. Accordingly, the total number of newly infected adults computes to (1 − (1 − *t*_*V*_)^*c*^)$\tilde{U}$. These suffer from a reduced survival rate of (1 − *s*) in comparison to uninfected individuals or individuals with “old” infections. As a result, there are $\tilde{I}$ + (1 − *s*)(1 − (1 − *t*_*V*_)^*c*^)$\tilde{U}$ infected and (1 − *t*_*V*_)^*c*^$\tilde{U}$ uninfected adults in the population.

The fourth and last step is the density dependent regulation during larval development. We followed a standard model for mosquito population dynamics developed by Christopher Dye (1984), and assumed that the *L* larvae develop into *L* = *exp*(−α*L*^β^)adults. The parameters (α, β) describe the density dependent mortalities of the larvae. Over- and undercompensation occurs depending on the choice of the parameters and the actual value of the variables (*I*, *U*).

We are now able to write down the full recursion equations. It holds that:

(2) $$U^{\prime }={\left(1-t_{V}\right)}^{c}\left(1-d_{A}\right)U+L\;\exp\left(-\alpha L^{\beta }\right)$$

(3) $$I^{\prime }=\left(1-d_{A}\right)I+\left(1-s\right)\left(1-{\left(1-t_{V}\right)}^{c}\right)\left(1-d_{A}\right)U,$$

where *L* = ε(1 − *d*_*L*_)(*I* + *U*) and *c* = (1 − *d*_*A*_) *I*/*K*.

### Model analysis

**Figure 2:**
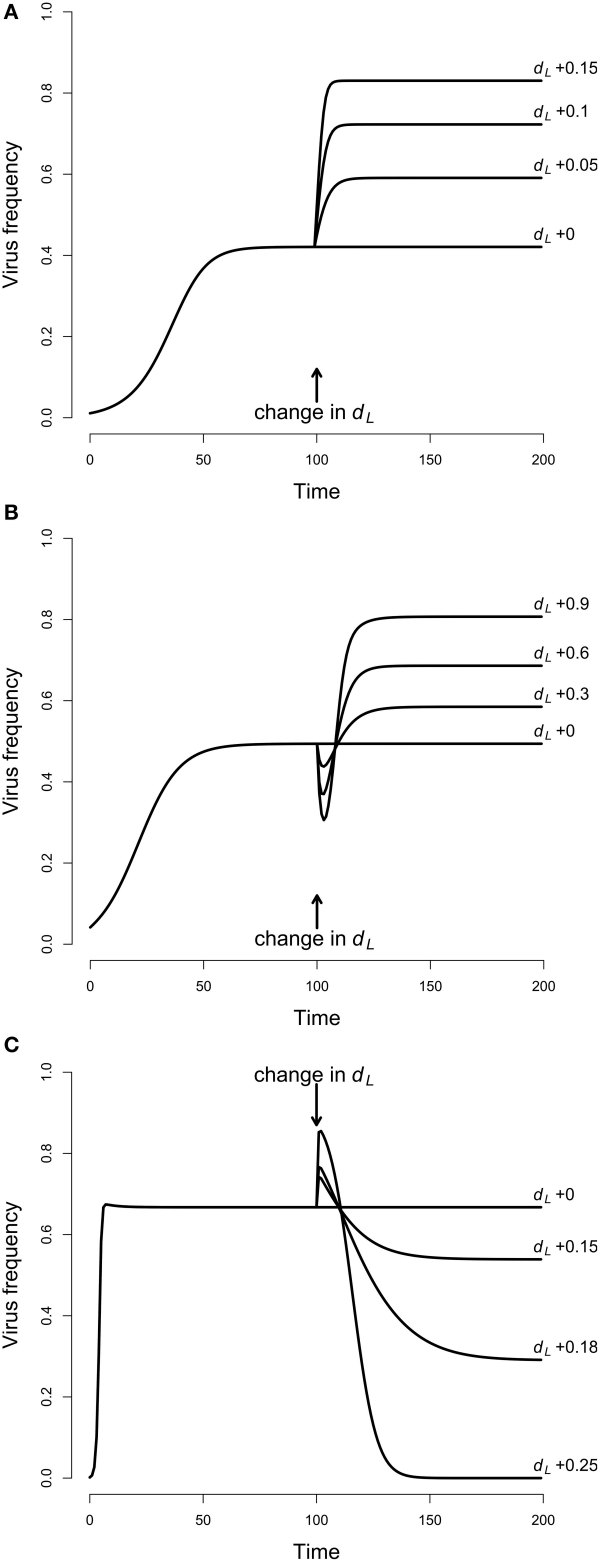
**Temporal dynamics of virus frequencies**. The virus was introduced to the host population with low frequency of 1% and then allowed to reach equilibrium. At generation 100 larval mortality *d*_*L*_ was increased and changes in virus frequencies were observed. **(A)** Adult competition model (ACM). The larval mortality *d*_*L*_ = 0.8 was increased by 0.05, 0.1, and 0.15. This resulted in increased virus frequencies, and highest equilibrium values were reached for highest larval mortality. Parameters: *d*_*A*_ = 0.1, ε = 1, *s* = 0.2, *t*_*V*_ = 0.95. **(B)** Larval competition model (LCM) with overcompensation. The larval mortality, starting with *d*_*L*_ = 0 was increased by 0.3, 0.6, and 0.9. The virus frequencies declined first, and increased afterwards up to high equilibrium values. Strongest effects occurred for the largest *d*_*L*_. Parameters: α = 0.15, β = 0.6, *d*_*A*_ = 0.1, ε = 15, *s* = 0.1, *t*_*V*_ = 0.95, *K* = 100. **(C)** LCM with undercompensation. The larval mortality *d*_*L*_ = 0.7 was increased by 0.15, 0.18, and 0.25. This resulted in virus dynamics opposite to **(B)**, i.e., there is first an increase and then a decrease of the virus frequencies. Lowest equilibrium frequencies occurred for largest *d*_*L*_. Parameters: α = 0.1, β = 0.4, *d*_*A*_ = 0.1, ε = 4, *s* = 0.1, *t*_*V*_ = 0.95, *K* = 100.

**Figure 3:**
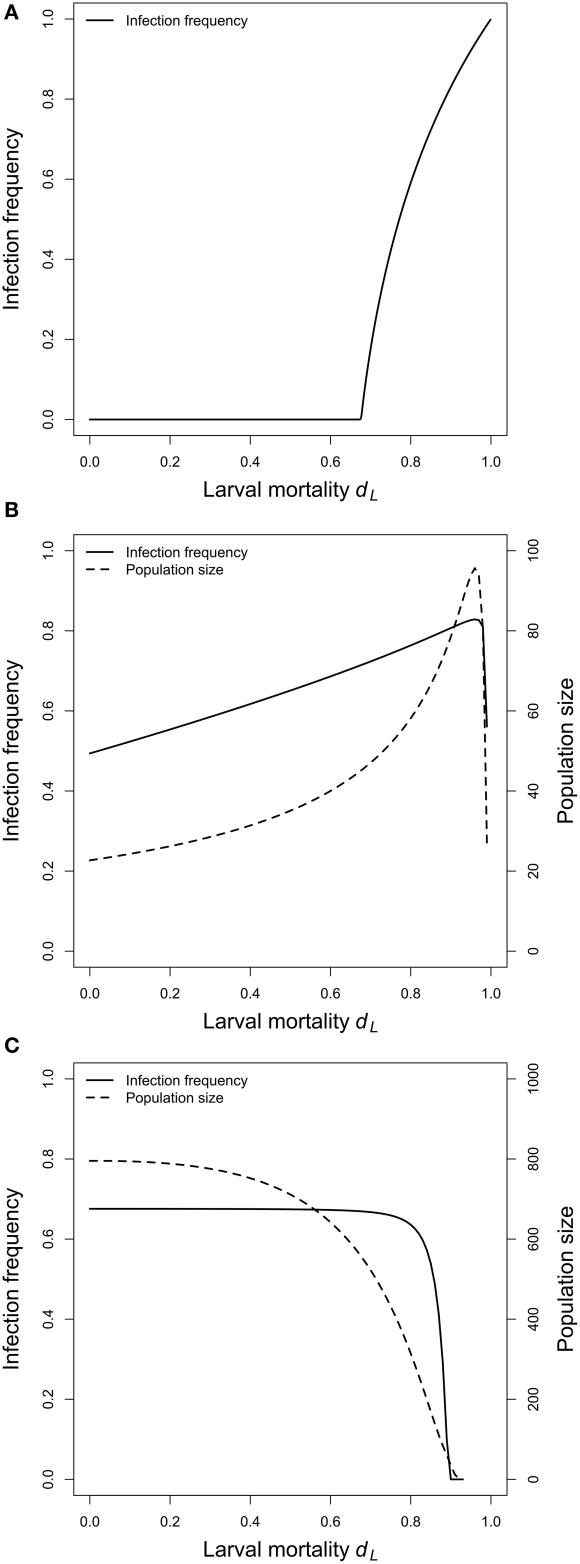
**Virus equilibrium frequencies and equilibrium population sizes. (A)** Adult competition model (ACM). Shown is the equilibrium frequency of the virus as a function of the larval mortality *d*_*L*_. There is a threshold value of *d*_*L*_, below which the virus cannot persist in the population. Above this critical value, virus frequencies increase with increasing larval mortality. **(B)** Larval competition model (LCM) with overcompensation. Shown are the equilibrium values of the infection frequency and the population size as a function of *d*_*L*_. The equilibria increase with increasing larval mortality, and reach maximum values at *d*_*L*_ = 0.96. Larger *d*_*L*_ results in a sharp decline of both values. **(C)** LCM with undercompensation. Infection frequencies and adult population sizes decrease with increasing larval competition. **(B,C)** show that the adult population size is the main determinant of the infection frequencies. Parameters: see Figure 2.

**Figure 4:**
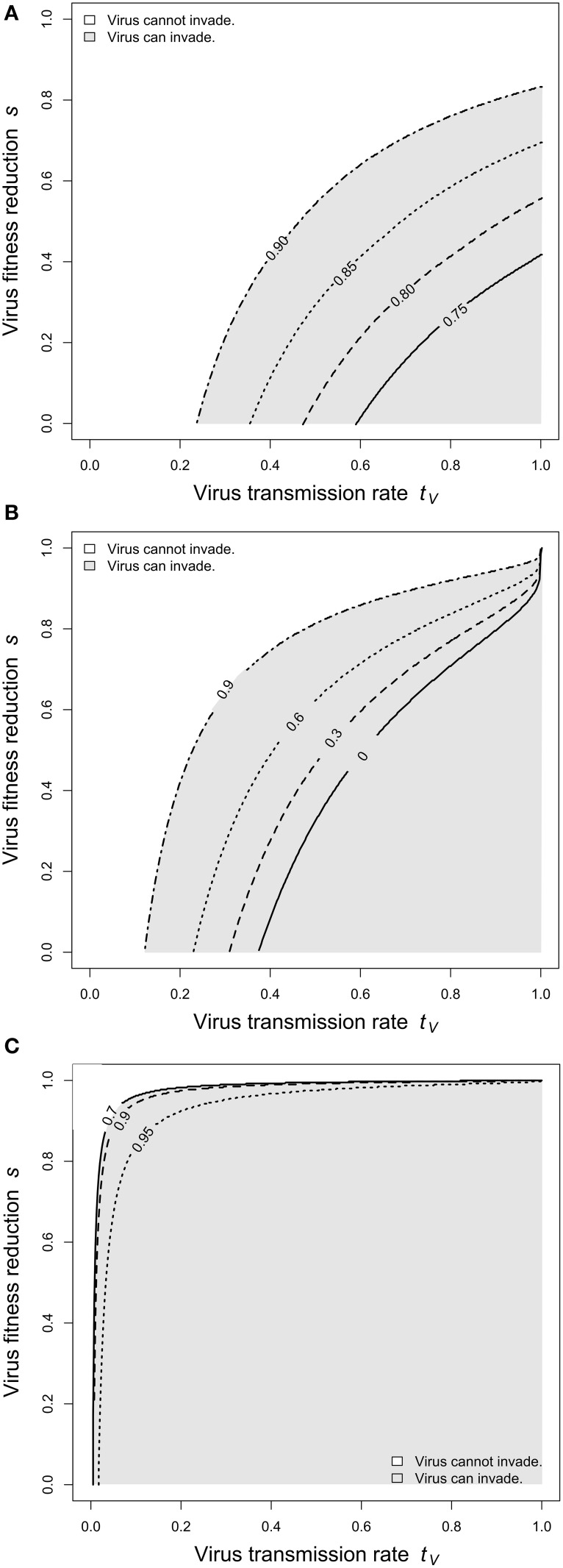
**Virus invasibility for varying levels of larval competition**. The parameter space spanned by virus transmission (*t*_*V*_) and cost of infection (*s*) was screened for parameter regions, in which the virus can invade the host population. **(A)** Adult competition model. Lines indicates *d*_*L*_ = 0.7 (solid), *d*_*L*_ = 0.8 (dashed), *d*_*L*_ = 0.85 (dotted), and *d*_*L*_ = 0.9 (dashed-dotted). **(B)** Larval competition model overcompensation. Lines indicates *d*_*L*_ = 0 (solid), *d*_*L*_ = 0.3 (dashed), *d*_*L*_ = 0.6 (dotted), and *d*_*L*_ = 0.9 (dashed-dotted). **(C)** Larval competition model undercompensation. Lines indicates *d*_*L*_ = 0.7 (solid), *d*_*L*_ = 0.9 (dashed), and *d*_*L*_ = 0.95 (dotted). The figure shows that high levels of larval competition facilitate virus invasibility for the ACM and the LCM with overcompensation, but impede invasibility for the LCM and undercompensation. Further parameters are as in Figure 2.

The ACM and the LCM were implemented as *Python 2.7.1* scripts and visualized with *Gnu R*. The ACM was analyzed by numerically iterating equation (1) (cf. Figure 2A). Virus equilibrium frequencies were calculated by iterating equation (1) until frequency changes between subsequent time steps were less than 10^−5^ (cf. Figures 3A, 4A). This procedure was conducted for high (99%) and low (1%) initial frequencies of the virus. For all parameters tested, the different starting conditions yielded the same equilibrium values (i.e., differed by less than 10^−5^). The LCM was analyzed by iterating Equations (2) and (3). For each parameter constellation tested, the system was first iterated 1000 time steps without the virus to avoid population growth effects. Then, one single individual was infected. Temporal dynamics and virus equilibria were calculated in the same way and with the same accuracy as the ACM (cf. Figures 2B,C, 3B,C, 4B,C).

## Results

### Adult competition model

In a first step, we investigated the temporal dynamics (Figure 2A). The virus was introduced to the system with the low frequency of 1% and allowed to reach equilibrium. After 100 generations, larval mortality (*d*_*L*_) was increased to mimic the effect of *Wolbachia*. The system was again allowed to reach equilibrium, and the equilibrium values before and after the change in *d_*L*_* were compared. For all parameters tested, an increase in *d_*L*_* resulted in increased viral frequencies. For example, a *d_*L*_* of 0.4 led to a virus frequency of 42%. However, if *d_*L*_* was increased to 0.45, then the equilibrium frequency was at 59%, and an increase to 0.55 resulted in the high virus frequency of 83% (Figure 2A). A parameter screen showed more generally that virus equilibrium frequencies increase with increasing larval mortality (Figure 3A). This effect may seem counter-intuitive but is actually expected. In the ACM, competition happens between adults and larvae. Accordingly, if larval mortality is increased, then the strength of competition is reduced. As a consequence, all adults (infected and uninfected) have a higher survival rate and therefore a higher chance to reach the next generation. Because infected adults serve as a reservoir for the virus, the overall virus frequency among adults is increased.

Next, we investigated how changes in larval mortality affect virus invasibility. In general, virus spread and persistence is promoted by high horizontal transmission (*t_*V*_*) and low cost of infection (*s*). We screened the (*t_*V*_*, *s*)-space for regions, in which the virus can invade the host population. Figure 4A shows that this region increases substantially with increasing *d_*L*_*.

In summary, increasing larval mortality has two effects: (1) virus frequencies are increased (Figures 2A, 3A), and (2) invasion of viruses is facilitated (Figure 4A). *Wolbachia* infections that increase larval mortality may therefore *indirectly* affect the virus dynamics in both ways even if *Wolbachia* and the virus do not interfere directly in coinfected host individuals (see also discussion, Supplementary Material).

### Larval competition model

For the LCM, two cases need to be distinguished, overcompensation and undercompensation. For overcompensation, increased larval mortality results in increased virus frequencies at equilibrium. For example, *d_*L*_* of 0.3, 0.6, and 0.9 lead to virus equilibrium frequencies of 58, 69, and 81% (Figure 2B). This is qualitatively similar to the adult competition model. In contrast to the ACM, however, the virus frequencies do not increase monotonously, but first decrease for several generations before they reach high equilibrium values. This is explained as follows. For overcompensation, lowering *d_*L*_* results in a higher number of newly emerging adults, which leads to a higher adult population size. In the short run, virus frequencies decrease because all newly emerging adults are uninfected. In the long run, however, the increased population size leads to a higher contact rate. This promotes virus transmission and leads to increased virus frequencies. The latter point is illustrated in Figure 3B. As evident from the graph, equilibrium virus frequencies increase with increasing larval mortality up to *d*_*L*_ = 0.96. This increase is accompanied by an increase in the adult population size. Note that for *d_*L*_* > 0.96, the system switches from over- to undercompensation, and both virus equilibrium frequency and adult population size decline sharply.

Next, we investigated virus invasibility. Figure 4B shows that an increase in larval competition enlarges the parameter space, for which viruses can spread. Biologically, this means that certain viruses (e.g., with low transmission rate or high costs for the host) go to extinction if larval competition is weak, but can spread and persist in the host population if *d_*L*_* is low. In a sense, strong larval competition makes the host vulnerable to viral infections. This result is qualitatively the same as described above for the ACM (cp. Figure 4A).

In contrast to the LCM with overcompensation, however, the model with undercompensation shows the opposite results. First, after an increase of *d_*L*_*, there is a short time, in which virus frequencies increase (Figure 2C). Subsequently, virus frequencies decrease and reach equilibrium values below the initial virus frequencies (Figures 2C, 3C). Second, virus invasion is impeded by strong larval competition (Figure 4C). These results are explained in the same way as above if taken into consideration that, for undercompensation, an increase in larval competition results in a lower number of emerging adults and a reduced adult population size.

In conclusion, changes in larval competition have opposite effects on the virus depending on the host life cycle. In general, a reduction in larval competition impedes spread and persistence of a virus in the LCM with undercompensation, but promotes it for the ACM and the LCM with overcompensation.

## Discussion

In this study, we investigated the effect of *Wolbachia* on the infection dynamics of horizontally transmitted viruses. In contrast to previous models, we considered a scenario, in which *Wolbachia* and the virus do not interfere directly inside single host individuals. Our key result is that *Wolbachia* affect the virus dynamics *indirectly* because *Wolbachia*-induced reproductive phenotypes (MK or CI) reduce larval density in infected populations. The mathematical analysis revealed that the outcome of the indirect effect is sensitive to the host's life history. The findings suggest that the spread of *Wolbachia* results (1) in reduced virus prevalence for host populations with density dependent regulation at the larval stage and undercompensation, and (2) in increased virus prevalence for host populations with density dependent regulation at the adult stage or larval competition and overcompensation.

Above, we modeled the effect of *Wolbachia* as a reduction in larval mortality without taking into account the infection dynamics of *Wolbachia* itself. This approach was chosen to emphasize that MK and CI phenotypes have a similar effect on the virus dynamics. However, we also investigated an extended version of the ACM that describes explicitly the coinfection dynamics of the virus with *Wolbachia* (either MK or CI). The following general conclusions can be made (see supplement for details). First, the results revealed that, as MK frequency increases, the virus frequency increases, and achieves its maximum for the highest MK frequency (Figure S1A). This is explained by the indirect effect under the assumption that larval mortality increases monotonously with MK frequencies. Second, the virus frequency increases during the spread of CI-*Wolbachia*, and has a pronounced peak when 50% of the population is infected with *Wolbachia*. High frequencies of *Wolbachia*, however, result only in a moderate increase of the virus, and if all individuals are infected with *Wolbachia*, there is no increase in virus frequency (Figure S1B). These results are also explained by the indirect effect. In contrast to MK, larval mortality is highest for CI if *Wolbachia* frequencies are around 50%.

An important question is whether these theoretical predictions are relevant for natural systems. Field studies suggest that *Wolbachia* can substantially reduce larval density in their arthropod hosts. Here, we discuss two examples in detail. First, high MK frequencies of 25% up to ~100% were reported in females butterflies of *Hypolimnas bolina* (Charlat et al., 2005). This results in 12.5–50% reduction of larvae because all infected males die in early embryogenesis. Second, intermediate frequencies of CI-*Wolbachia* were reported for the two-spotted spider mite *Tetranychus urticae*. Large scale screening in 17 populations revealed that infection frequencies range from 2.5 to 77.5% with a median of 27.5% (Chen et al., 2009; Yu et al., 2011; Su et al., 2012). Under the assumption of random mating, a *Wolbachia* frequency of *p* is expected to reduce the population wide hatch rate by (1-*p*)*pl*_*CI*_, where *l*_*CI*_ denotes the CI-level (i.e., proportion of offspring that die in an incompatibility mating). Accordingly, a parameter constellation of *l*_*CI*_ = 0.5 and *p* = 27.5%, which is realistic for *T. urticae* (Gotoh et al., 2007), causes 10% reduction in hatch rate. However, the reduction can be as high as 25% if *l*_*CI*_ = 1 and *p* = 50%. In conclusion, both MK and CI may substantially reduce larval density in natural populations. This argues for a significant role of the *indirect effect* in arthropod populations that are coinfected by *Wolbachia* and horizontally transmitted viruses.

Our results may have important implications for pathogen ecology and pest control. We showed that the presence or absence of *Wolbachia* determines whether certain viruses can persist in the host or go to extinction (Figure 4). Phylogenetic comparisons between *Wolbachia* and host species, however, indicate a frequent gain and loss of *Wolbachia* infections on an evolutionary time scale (Malloch and Fenton, 2005; Viljakainen et al., 2008; Zug and Hammerstein, 2012). Once *Wolbachia* enters a new host population, it can spread rapidly up to high infection frequencies (Turelli and Hoffmann, 1991), and losses may also happen in rather short time (Koehncke et al., 2009). Our findings suggest that the pathogen community of a host species may substantially change with every single *Wolbachia* gain or loss. Given the wide distribution of *Wolbachia* in terrestrial arthropods, *Wolbachia* might be an important player in the ecology of arthropod associated viruses.

There is a growing interest in using *Wolbachia* as a means for controlling insect pests and disease vectors. This is because *Wolbachia* is, first, able to spread rapidly in new host populations and, second, has the potential to impede pathogen replication inside the vector. Our theoretical analysis suggests that the host life cycle may be an important factor for the success or failure of such biocontrol programs. Vectors like mosquitoes may be especially difficult to control because over- and undercompensation can occur in the same species, but under different environmental conditions as reported for the dengue vectors *Ae. aegypti* and *Ae. albopictus* (Walsh et al., 2011). Accordingly, a biocontrol program may be successful only in certain geographical regions or at certain time in the year. This argues for the need to carefully test for over- and undercompensation before and during the release of *Wolbachia* to natural populations.

Previous theoretical analysis demonstrated a *direct effect* of *Wolbachia* on viruses that results in reduced virus frequencies if *Wolbachia* impedes virus replication in coinfected host individuals (Fenton et al., 2011). This effect is intuitive. Our analysis demonstrated a counter-intuitive *indirect effect* of *Wolbachia* on the virus that occurs because *Wolbachia* infections alter either the age structure (ACM) or the population size (LCM) of the host. The indirect effect was not observed by Fenton et al. (2011). It would be interesting to analyze an extended model that incorporates the basic features of Fenton's and our ACM/LCM model. We expect that the virus dynamics in such a model is shaped by both, the direct and the indirect effect, and that the two effects either amplify or cancel each other out, depending on the host's life history. Complex effects may occur for mosquitoes because their population dynamics, as discussed above, may change in space and time between over- and undercompensation depending on environmental conditions.

We have pointed out that the host life-cycle A previous theoretical study by Martinez-Rodriguez et al. (2014) pointed out that the host life cycle can alter the infection dynamics of CI-inducing *Wolbachia*, e.g., if some host individuals loose the infection during their life span. An important implication of this study is that hybrid zones involving uni- and bidirectional CI can stably persist, which would not be possible according to standard models (cf. Engelstädter and Telschow, 2009). In our analysis, we did not consider a possible loss of *Wolbachia* during the host life cycle. It may be an interesting direction for future research to study how such a *Wolbachia* loss affects the infection dynamics of both *Wolbachia* and the virus.

In summary, our results show that *Wolbachia* affect the infection dynamics of viruses even if *Wolbachia* does not interfere with the virus directly in coinfected hosts. This indirect effect of *Wolbachia* can result in increased or decreased virus frequencies depending on the host's life cycle. These findings point out that the usability of *Wolbachia* in biocontrol programs depends crucially on the specific host-virus system.

### Conflict of interest statement

The authors declare that the research was conducted in the absence of any commercial or financial relationships that could be construed as a potential conflict of interest.
