# Supplementary material for: Modeling the indirect effect of Wolbachia on the infection dynamics of horizontally transmitted viruses
Source: Front Microbiol. 2015 Apr 28;6:378. doi: 10.3389/fmicb.2015.00378 (PMC4412059; doi:10.3389/fmicb.2015.00378)
Supplement: Supplementary file 1 [file DataSheet1.PDF]

Supplementary Material

Modeling the indirect effect of Wolbachia on the infection Dynamics of horizontally transmitted viruses.

Jakob Friedrich Strauß[1], Arndt Telschow[2]*

[1]Institute for Evolution and Biodiversity, Westfälische Wilhelms Universität, Münster, Germany

*** Correspondence:** Arndt Telschow, Institute for Evolution and Biodiversity, Westfälische Wilhelms Universität Münster, Hüfferstraße 1, Münster, D-48143, Germany.
a.telschow@uni-muenster.de

Supplementary Data

1. Supplementary model and figures.

Model with explicit *Wolbachia* frequency.

We designed a population genetics model in order to describe the dynamics of Wolbachia bacteria and a generalized horizontally transmitted virus. The model is deterministic and assumes an infinite population. It has discrete time steps. We consider two model variations for *Wolbachia*. Male-killing and cytoplasmic incompatibility. A summary of the model parameters can be found in the following table:

Table 1: Model parameters

Parameter	Description
$\boldsymbol{t_B}$	*Wolbachia* transmission rate
$\boldsymbol{b}$	fitness compensation
$\boldsymbol{l_{CI}}$	level of cytoplasmic incompatibility
$\boldsymbol{s}$	viral fitness reduction
$\boldsymbol{t_h}$	horizontal virus transmission rate
$\boldsymbol{d}$	larval mortality
$\boldsymbol{a}$	interaction rate

We assume an arthropod host for the *Wolbachia* and the virus that has discrete, but overlapping generations. The basic *Wolbachia*/virus model describes the change in infection frequency. It consists of a $X_{\vec{\imath}}$ that describes all frequencies depending on the state of gender, virus infection and MK infection. The vector $\vec{\imath}$ consists of i_1, i_2, i_3 with i_1 being the gender (0 female, 1 male), i_2 being the virus infection state (0 uninfected, 1 infected) and i_3 the MK infection state (0 uninfected, 1

infected). All other vectors like $\vec{n}$ or $\vec{j}$ are used accordingly. T denotes the weighting factor for the viral infection. J denote the weighting factor for the bacterial infection. B denotes the weighting factor for the fitness compensation and $\widetilde{D}$ and D the weighting factors for the virus induced and MK induced death rates. In the case of CI the weighting factor $\widetilde{D}$ and B is switched with the weighting factor CI. The weighting factors weight the proportion of offspring that goes into the frequencies of the next generation.

The model with explicit *Wolbachia* frequencies follows a simple life cycle: (1) Reproduction and vertical inheritance of *Wolbachia*, (2) density control, (3) horizontal virus transmission, (4) density control.

(1) Reproduction and vertical inheritance of Wolbachia.

The first step consists of mating and reproduction, adult mortality, and maternal inheritance of *Wolbachia*.

(1.A) Male-killing:

For the MK part of the model we follow Hurst (1991) and Randerson et al. (2000) and describe the dynamics by two parameters, the bacterial transmission rate t_B and the fitness compensation b. MK infected females transmit *Wolbachia* maternally over the egg cytoplasm to the fraction t_B of their offspring. Daughters as well as non MK-infected sons profit from MK infected brothers by the fitness compensation factor b, due to an advantage in resource competition. We assume perfect effectivity in MK, which means that all infected males die in an early embryonic stage. As a result, there are no MK infected males in the population. Note that a hidden assumption of the model is a limited male mating capacity. As a consequence, not every female gets a mating partner if MK frequencies are high. The frequencies after the first step in the life cycle compute to

$$X_{\vec{\imath}}^{+} = X_{\vec{\imath}} D_{i_2} + (1-d)\widetilde{D}_{\vec{\imath}} \sum_{j_2,j_3,n_2,n_3}^{1} \left(X_{0,j_2,j_3} X_{1,n_2,n_3} J_{i_3,j_3} D_{i_2} B_{j_3}\right).$$

(1.B.) Cytoplasmic incompatibility:

We altered the model by exchanging male-killing with cytoplasmic incompatibility. In the case of CI we drop the weighting factors J and B and add the weighting factor CI. With cytoplasmic incompatibility we modify the offspring by a factor l_{CI}, if we have an incompatibility match, i.e. uninfected female X infected male. If enough infected males are present, the fitness reduction of uninfected females, caused by incompatibility matches, drives the spread of the CI *Wolbachia* infection. The frequencies after the first step in the life cycle compute to

$$X_{\vec{\imath}}^{+} = X_{\vec{\imath}} D_{i_2} + (1-d)\widetilde{D}_{\vec{\imath}} \sum_{j_2,j_3,n_2,n_3}^{1} \left(X_{0,j_2,j_3} X_{1,n_2,n_3} CI_{i_3,j_3} D_{i_2}\right).$$

(1.C) The weighting factors:

Fitness compensation only happens in the case of male-killing. The weighting factor for the fitness compensation is defined as follows:

$$B_{j_3} = \begin{cases} 1 & j_3 = 0 \\ 1 + b\, t_B & j_3 = 1 \end{cases}$$

Cytoplasmic incompatibility only happens in the presence of CI-*Wolbachia*. The weighting factor CI is defined as follows (i offspring, n male parent, j female parent):

$$CI_{i_3,n_3,j_3} = \begin{cases} 1 & i_3 = 0 \quad n_3 = 0 \quad j_3 = 0 \\ 1 - t_B & i_3 = 0 \quad n_3 = 0 \quad j_3 = 1 \\ l_{CI} & i_3 = 0 \quad n_3 = 1 \quad j_3 = 0 \\ 0 & i_3 = 1 \quad n_3 = 0 \quad j_3 = 0 \\ t_B & i_3 = 1 \quad n_3 = 1 \quad j_3 = 0 \\ 0 & i_3 = 1 \quad n_3 = 1 \quad j_3 = 0 \\ (1 - t_B) l_{CI} & i_3 = 0 \quad n_3 = 1 \quad j_3 = 1 \\ t_B & i_3 = 1 \quad n_3 = 0 \quad j_3 = 1 \end{cases}$$

The weighting factor for the MK induced mortality is denoted as follows:

$$\widetilde{D}_{\vec{\imath}} = \begin{cases} 0 & i_1 = 1, i_3 = 1 \\ 1 & \text{else} \end{cases}$$

The weighting factor for the virus-induced mortality is defined as follows:

$$D_{i_2} = \begin{cases} 1 & i_2 = 0 \\ 1 - s & i_2 = 1 \end{cases}$$

The weighting factor for MK describes both the vertical inheritance and the male-killing.

$$J_{i_3,j_3} = \begin{cases} t_B & i_3 = j_3 = 1 \\ 1 - t_B & i_3 = 0, j_3 = 1 \\ 1 & i_3 = j_3 = 0 \\ 0 & i_3 = 1, j_3 = 0 \end{cases}$$

(2) Density control.

After (1) X is normalized to the sum of all intermediate frequencies. This way all female frequencies add up to 1, as do the male frequencies:

$$X_{\vec{\imath}}^{++} = X_{\vec{\imath}}^{+} \left(\sum_{j_2,j_3}^{1} X_{i_1,j_2,j_3}^{+} \right)^{-1}$$

(3) Horizontal virus transmission.

For the horizontal transmission we introduce a round of infection after the normalization. First we add a proportion of previously uninfected frequencies to the infected frequencies, and subtract the newly infected from the respective sequences. Uninfected can get infected by having interactions at the rate a with the proportion of infected adults times the transmission probability t_h. D_h is a second

round of mortality for the newly infected.

$$X^{+++}_{i_1,1,i_2} = X^{++}_{i_1,1,i_3} + D_h\, a\, X^{++}_{i_1,0,i_3}\, \frac{1}{2}\left(X^{++}_{i_1,1,1} t_h + X^{++}_{0,1,0} t_h + X^{++}_{1,1,0} t_h\right)$$

$$X^{+++}_{i_1,0,i_2} = X^{++}_{i_1,0,i_3} - a\, X^{++}_{i_1,0,i_3}\, \frac{1}{2}\left(X^{++}_{i_1,1,1} t_h + X^{++}_{0,1,0} t_h + X^{++}_{1,1,0} t_h\right)$$

(4) Density control.

We finish the life cycle by normalizing for a second time:

$$X^{++++}_{\vec{\iota}} = X^{+++}_{\vec{\iota}} \left(\sum_{j_2,j_3}^{1} X^{+++}_{i_1,j_2,j_3}\right)^{-1}$$

1.1 The indirect effect of male-killing *Wolbachia* on the virus.

We screened the *Wolbachia* transmission rate t_B ($0.7 \leq t_B < 1$) for the *Wolbachia* equilibrium $\boldsymbol{W}^*$and the virus equilibrium. When the male-killing *Wolbachia* can invade ($t_B > 0.86$), both the sex-ratio and the virus equilibrium change (see fig. S1-A). The sex-ratio shifts towards females, depending on how far the male-killing *Wolbachia* can spread, as does the virus equilibrium. In this model, the frequency of the *Wolbachia* infection determines the increase in larval mortality. As we assume perfect effectivity for male-killing, the increase in larval mortality can be characterized as $\Delta d = \frac{\boldsymbol{W}^*}{2} d$. The indirect effect described in the main text applies for this increase in larval mortality.

1.2 The indirect effect of cytoplasmic incompatibility causing *Wolbachia* on the virus.

We screened the *Wolbachia* transmission rate t_B for the *Wolbachia* equilibrium $\boldsymbol{W}^*$ and the virus equilibrium. When the cytoplasmic incompatibility inducing *Wolbachia* can invade ($t_B > 0.82$), the virus equilibrium reaches its maximum at the lowest *Wolbachia* frequency. In theory the largest change in larval mortality should be caused by *Wolbachia* at 50% frequency, as this would maximize the frequency of incompatibility matches. The increase in larval mortality can be characterized as $\Delta d = (1 - \boldsymbol{W}^*)\, l_{CI}\, d$. When *Wolbachia* reaches 100%, there are no incompatibility matches and the virus reaches the exact equilibrium it had before *Wolbachia* invaded. The indirect effect described in the main text applies for this increase in larval mortality.

1.4 Supplementary Figures

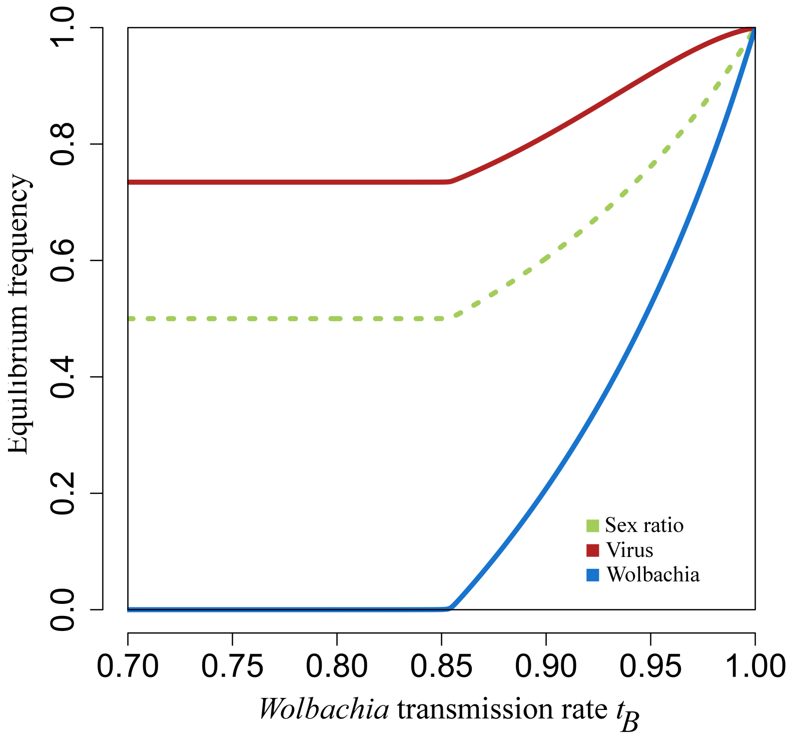

Supplementary Figure 1-A. Equilibirum frequencies of male-killing *Wolbachia* and a virus. We show the *Wolbachia* equilibrium (blue) and the virus equilibrium (red) for the model with explicit Wolbachia frequencies. The equilibria are shown for different rates of the *Wolbachia* transmission rate $\boldsymbol{t_B}$(0.7 .. 1). Paramters were set as follows: $\boldsymbol{b = 0.2, t_h = 0.9, s = 0.2}$ and $\boldsymbol{d = 0.7}$. For this simulation we first let the virus go to equilibrium ($\Delta < \mathbf{10^{-5}}$), and subsequently introduced a *Wolbachia* infection at 1% infection frequencies. We then let the simulation run till both the virus and the *Wolbachia* frequencies stopped changing ($\Delta < \mathbf{10^{-5}}$). The green dashed line indicates the sex ratio, as percentage of females.

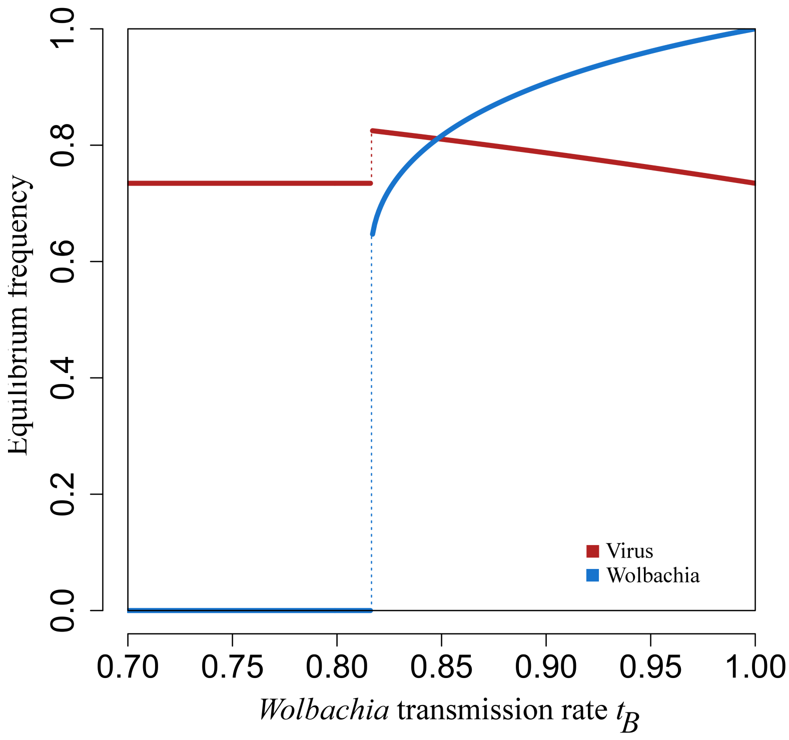

Supplementary Figure 1-B. Equilibirum frequencies of cytoplasmic incompatibility *Wolbachia* and a virus. We show the *Wolbachia* equilibrium (blue) and the virus equilibrium (red) for the model with explicit Wolbachia frequencies. The equilibria are shown for different rates of the *Wolbachia* transmission rate $\boldsymbol{t_B}$(0.7 .. 1). Paramters were set as follows: $\boldsymbol{l_{CI} = 0.6, t_h = 0.9, s = 0.2}$ and $\boldsymbol{d = 0.7}$. For this simulation we first let the virus go to equilibrium ($\Delta < \mathbf{10^{-5}}$), and subsequently introduced a *Wolbachia* infection at 1% infection frequencies. We then let the simulation run till both the virus and the *Wolbachia* frequencies stopped changing ($\Delta < \mathbf{10^{-5}}$).
