# Supplementary material for: Modeling the indirect effect of Wolbachia on the infection dynamics of horizontally transmitted viruses
Source: Front Microbiol. 2015 Apr 28;6:378. doi: 10.3389/fmicb.2015.00378 (PMC4412059; doi:10.3389/fmicb.2015.00378)
Supplement: Supplementary file 2 [file DataSheet2.PDF]

Supplementary Material II

Modeling the indirect effect of Wolbachia on the infection Dynamics of horizontally transmitted viruses.

Jakob Friedrich Strauß[1], Arndt Telschow[2]*

[1]Institute for Evolution and Biodiversity, Westfälische Wilhelms Universität, Münster, Germany

*** Correspondence:** Arndt Telschow, Institute for Evolution and Biodiversity, Westfälische Wilhelms Universität Münster, Hüfferstraße 1, Münster, D-48143, Germany.
a.telschow@uni-muenster.de

Supplementary: Python implementation of ACM & LCM

If run, both scripts will output a simple table which gives time step and infection frequency.

1. Adult competition model

```
#!/usr/bin/python
# Adult Competition Model
import sys
from math import *

def next(I,da,dl,s,t):
    pI = (I*(1-da)+t*(1-s)*(1-da)**2*I*(1-I))/(I*(1-da)+t*(1-s)*(1-da)**2*I*(1-I)+(2-I-
dl)*(1-da)-t*I*(2-I-dl)*(1-da)**2+(1-dl))
    return pI

def run():
    I  = 0.01; da = 0.1; dl = 0.75; s  = 0.2; t  = 0.95
    i = 0
    while(i < 200):
        I = next(I,da,dl,s,t)
        print i, I
        i += 1
    dl += 0.15
    while(i < 400):
        I = next(I,da,dl,s,t)
        print i, I
        i += 1
run()
```

2. Larval competition model

```
#!/usr/bin/python
# Larval competition model
import sys
from math import *

def next(U,I,da,dl,e,a,b,s,t):

    K = 100
```

```
    L   = e*(1-dl)*(I+U)
    c = (1-da)*I/K
    T = (1-t)

    pU = T**c * (1-da)*U + L*exp(-a*(L**b))
    pI = (1-da)*I + (1-s)*(1-T**c)*(1-da)*U

    return pU, pI

def run():
    U = 10; I = 0; da = 0.1; dl = 0; e = 15 # overcompensation
    a = 0.15; b = 0.6; t = 0.72; s = 0.1

    #U = 10; I = 0; da = 0.3; dl = 0.7; e = 4 # undercompensation
    #a = 0.1 ; b = 0.4; t = 0.72; s = 0.1

    i = 0
    while(i<100):
        U,I = next(U,I,da,dl,e,a,b,s,t)
        i += 1

    I = 1
    i = 0
    print i, I/(U+I)
    i = 1

    while(i <= 100):
        U,I = next(U,I,da,dl,e,a,b,s,t)
        print i, I/(U+I)
        i += 1

    dl += 0.15

    while(i < 200):
        U,I = next(U,I,da,dl,e,a,b,s,t)
        print i, I/(U+I)
        i += 1

run()
```
